# Position statement on genuine physiotherapy research at German university hospitals

**DOI:** 10.3205/000332

**Published:** 2024-05-27

**Authors:** Susanne G. R. Klotz, Andrea Bökel, Maria Friderichs-Nedohibchenko, Isabelle Stickdorn, Barbara Vogel, Bernd Doods, Franziska Feldmann, Mirko Ghiazza, Markus Giehl, Annika Hoberg, Lynn Jansen, Daniel Kohlhofer, Ralf Leonhardt, Sebastian-Florian Meier, Carina Müller, Miriam Pannzek, Simone Schwarz, Martina Traut, Maria Urdahl, Network of Researching Physiotherapists at German University Hospitals

**Affiliations:** 1 Department of Physiotherapy, University Medical Center Hamburg-Eppendorf, Hamburg, Germany; 2 Department of Rehabilitation and Sports Medicine, Hannover Medical School, Hannover, Germany; 3 Department for Physical Medicine, Charité – Universitätsmedizin Berlin, Germany; 4 Department of Therapeutic Health Professions, University Hospital Münster, Germany; 5 Physical Therapy, Department of Orthopedics and Sports Orthopedics, University Hospital rechts der Isar, Technical University of Munich, Germany; 6 Central Department of Physiotherapy, University Medical Center Göttingen, Georg August University, Göttingen, Germany; 7 Institute for Physiotherapy, University Medical Center Essen, Germany; 8 Central Physiotherapy Department, University Medical Center Freiburg, Germany; 9 Department of Physiotherapy, University Hospital Würzburg, Germany; 10 Therapy Center, University Hospital Oldenburg AöR, Oldenburg, Germany; 11 Department of Physiotherapy, University Hospital RWTH Aachen, Germany; 12 Department of Physiotherapy and Occupational Therapy, University Hospital Augsburg, Germany; 13 Physical and Rehabilitative Medicine, University Hospital Erlangen, Germany; 14 Department of Physiotherapy and Occupational Therapy, University Hospital Regensburg, Germany; 15 Department of Physiotherapy, Ergotherapy, Logopedics and Physical Therapy, Universitätsmedizin Mannheim, Germany; 16 Department of Physiotherapy, Rostock University Medical Center, Rostock, Germany; 17 Central Facility for Physical Therapy and Rehabilitation, Leipzig University Hospital AöR, Leipzig, Germany; 18 Department of Physiotherapy, University Hospital Schleswig Holstein, Lübeck, Germany; 19 Department of Physiotherapy, University Hospital Schleswig Holstein, Kiel, Germany

**Keywords:** physiotherapy, research, university hospital, profession, genuine physiotherapy research

## Abstract

In addition to patient care, physiotherapy is increasingly important in research at university hospitals. Genuine physiotherapy research plays a decisive role in this. This position statement describes the opportunities, benefits, framework conditions, challenges, and research priorities of genuine physiotherapy research at German university hospitals.

## Abstract (de)

Die angehende Profession Physiotherapie nimmt neben der Patient:innenversorgung zunehmend eine wichtige Funktion in der Forschung im universitätsklinischen Setting ein. Die Entwicklung einer genuinen physiotherapeutischen Forschung spielt dabei eine entscheidende Rolle. Im Positionspapier werden Chancen und Nutzen, Rahmenbedingungen und Herausforderungen sowie Forschungsprioritäten der genuinen physiotherapeutischen Forschung an den deutschen Universitätskliniken dargestellt.

## Introduction

University hospitals are characterized by the combination of science and healthcare. In addition to their mandate to provide care to outpatients and inpatients at all levels of care, including maximum care, they also have a legal mandate to conduct scientific research and teaching [1]. Through this mission, they form the scientific foundation of the healthcare system. The interface between the scientific and healthcare systems can be seen as a double unique selling point: in the healthcare system, university hospitals differ from other institutions in that they are assigned scientific tasks, and in the scientific system, they differ from other institutions in that they take on healthcare tasks. However, science and healthcare do not run parallel to each other, but are ideally interlinked in terms of institutions, individuals, content, and infrastructure [2]. Research of university medicine aims to further develop and improve healthcare in terms of evidence-based, quality, effectiveness, and efficiency. In addition to preclinical basic research and clinical research, translational and healthcare research also have their place in the research portfolio of university hospitals. Research as one pillar in the classic task triad of university medicine is interrelated with the other two pillars of teaching and patient care. Science and healthcare can provide each other with recursive impulses, e.g. by transferring the generated research knowledge into practice, through transfer and practice projects or by taking up practical problems in research [2].

The emerging profession of physiotherapy is an important part of patient care in German university hospitals [3]. Physiotherapy in the acute medical setting has the potential to contribute to increasing the quality of life of patients [4], their satisfaction [5], and the physical activity level of patients [6], [7] as well as to a reduction in the length of hospital stay [8], [9].

Twenty-one of the 36 university hospitals have their own vocational school for physiotherapy [10] and currently address teaching as the second pillar of university medicine. Here, vocational training in physiotherapy takes place at level 4 of the German Qualifications Framework. However, the framework conditions for healthcare have changed: the complexity of care has risen continuously in recent decades, in part due to demographic change and the associated increase in people of advanced age, with chronic illnesses and multimorbidity, as well as digitalization and medical progress [11], [12], [13]. This leads to increased demands in care combined with the growing importance of interprofessional, interdisciplinary, and transdisciplinary collaboration [14], [15], [16], [17], [18], [19]. For this reason, German physiotherapy is striving to further develop its own profession and already embarked on the path to professionalization via academization to become a fully-fledged profession several decades ago [20], [21]. Higher education in its Bachelor’s, Master’s and doctoral qualification levels enables improved and high-quality patient care, as scientific evidence is transferred into practice [14], [17], [21], [22], [23], [24], [25], [26]. The establishment of degree programs can simultaneously strengthen the development and expansion of science, discipline formation and genuine research [27], [28], [29], [30]. 

Research can be understood as a prerequisite and adaptation to the changing framework conditions. It is therefore indispensable if physiotherapy wants to make its contribution to health-related issues in patient care, education and the profession [27], [28], [29], [30]. In their assessments of developments in the healthcare system, the German Science and Humanities Council [Wissenschaftsrat] and the German Expert Council for the Assessment of Developments in the Healthcare System [Sachverständigenrat zur Begutachtung der Entwicklung im Gesundheitswesen] have repeatedly called for physiotherapy to be more firmly established at medical faculties, including the expansion of research [2], [19], [31], [32].

**Figure 1:**
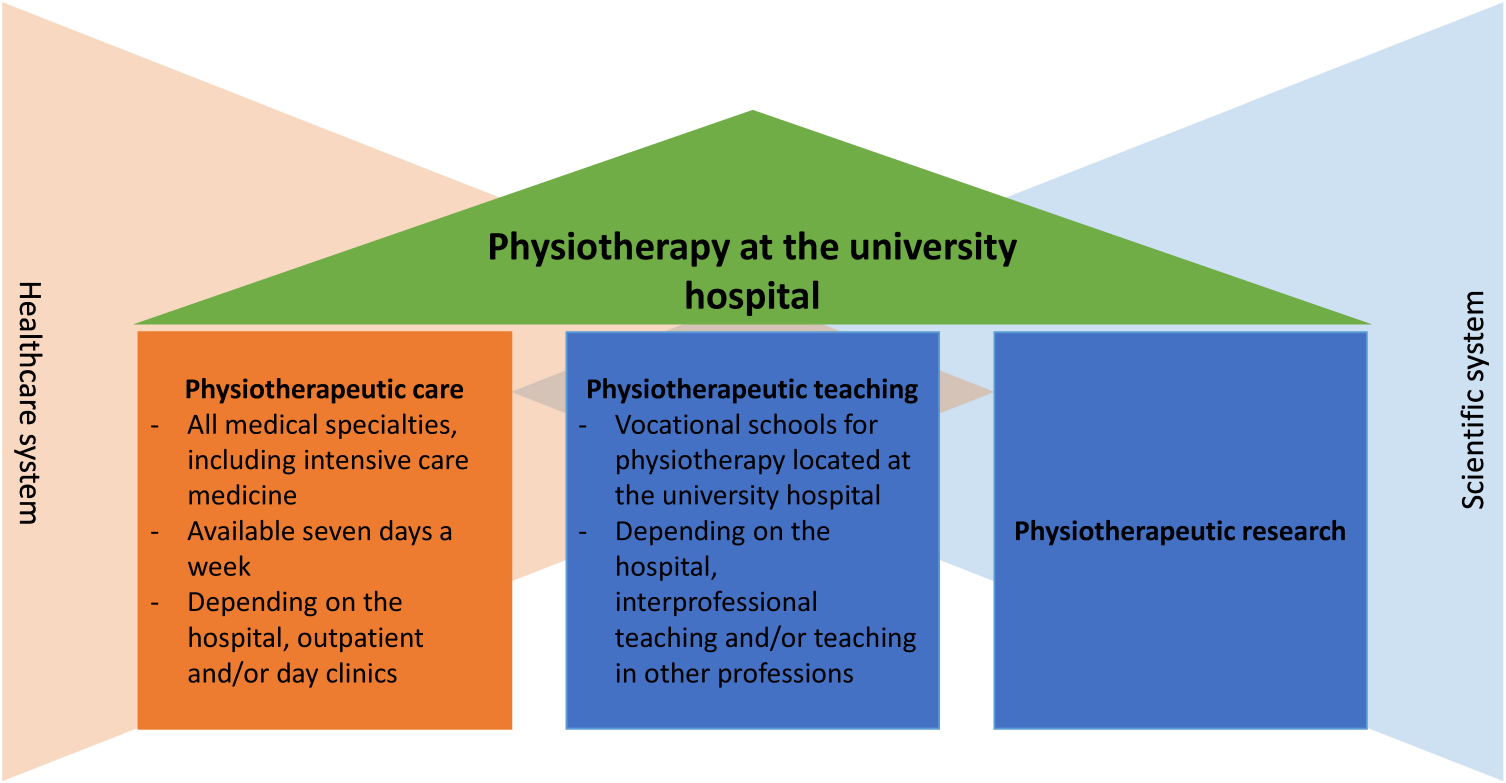
Three pillars of physiotherapy in the university hospital at the interface between the healthcare and scientific systems (own illustration based on [2])

With genuine research, physiotherapy could also represent the third pillar of university medicine (Figure 1 (Fig. 1)). In this context, the term genuine research is to be understood as original physiotherapeutic research carried out by physiotherapists from their own profession on physiotherapeutic issues. There is thus a distinction from research on physiotherapy carried out by other professions. Genuine research in physiotherapy already takes place at a few university hospitals, however, as recommended by the German Science and Humanities Council and the German Expert Council for the Assessment of Developments in the Healthcare System [2], [19], [31], it is hardly present in university medicine across the board. This position statement by the Network of Researching Physiotherapists at German University Hospitals therefore discusses the opportunities and benefits, framework conditions and challenges as well as research priorities of genuine physiotherapy research at German university hospitals.

## Opportunities and benefits

Genuine physiotherapy research at German university hospitals offers opportunities and benefits on various levels. The central benefits of physiotherapy research include quality assurance and improvement of patient care, the professionalization of the profession and the establishment of national and international cooperation in care and science. Due to their personnel and structural requirements, university hospitals in particular can create the basic framework conditions for successful physiotherapy research in accordance with their mission statements.

**Figure 2:**
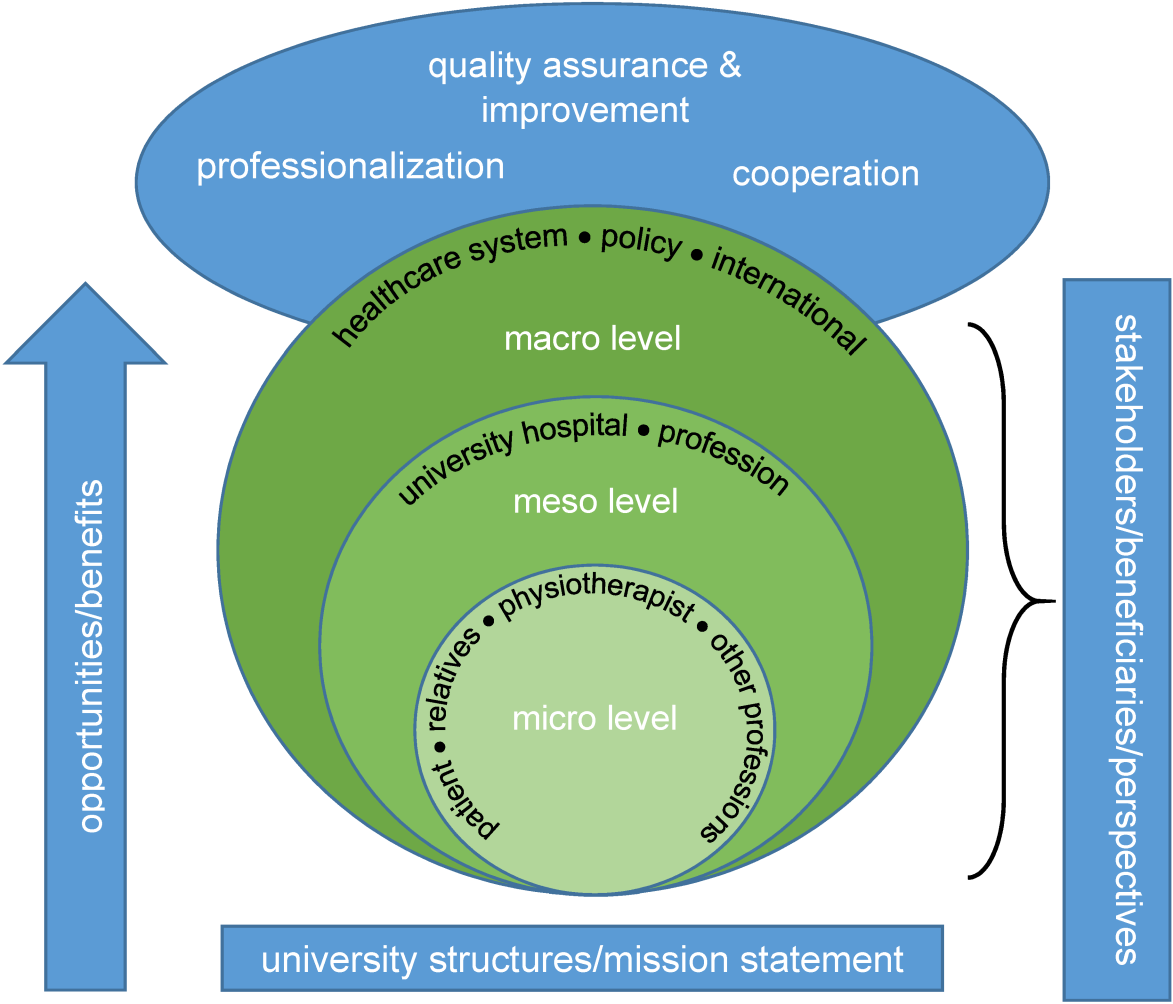
Opportunities and benefits of genuine physiotherapy research at German university hospitals at micro, meso and macro level

A large number of stakeholders benefit at the micro, meso and macro level of the healthcare system (Figure 2 (Fig. 2)). At the micro level, patients and their relatives, physiotherapists and other healthcare professionals should be named as beneficiaries of physiotherapy research. The meso level includes university hospitals and physiotherapy as a profession. The healthcare system, society and the international community are assigned to the macro level as stakeholders.

### Micro level

The primary benefit from the patient’s perspective is that a higher quality, modern and evidence-based therapy is guaranteed [27], [33], [34]. The involvement of patients in (physiotherapy) research is increasingly demanded and can contribute to improving the care situation of patients [35] in order to ensure needs-based, appropriate and economical care in accordance with paragraph 70 SGB V. The consideration of the patient’s perspective in the form of Patient Reported Experience and Outcome Measures (PREMs and PROMs) is of great benefit to genuine research [36], [37], [38], [39]. This strengthens the active patient role in the care context in terms of empowerment and adherence [40]. 

From the perspective of physiotherapists, genuine research offers the expansion of their own field of action combined with personal growth in knowledge and an increase in professional, methodological and practical competence [41]. As a result, the therapist’s job satisfaction and motivation can be positively influenced [42], [43]. Moreover, the expansion of the fields of action can lead to a more attractive financial appreciation and an increase in the attractiveness and prestige of the profession, among other things through the development of new career paths [44], [45]. The development of new academic career paths is strongly recommended [19], [32]. In their role as reflective practitioners and researchers, physiotherapists take responsibility and initiative for the further development and visibility of the profession [46], [47]. This includes the critical reflection of research results and their application in therapy [21], [48]. Therefore, patient safety can be improved and evidence-based care can be ensured, which has been demanded and promoted for years [49], [50].

In addition, genuine research at university hospitals offers the opportunity to improve interprofessional collaboration and care. This means that physiotherapists are perceived and recognized as cooperative, equal research partners. Interprofessional knowledge networking promotes the possibility of continuous mutual support and further development of care goals and their outcomes [51], [52].

### Meso level

By establishing physiotherapy research, university hospitals are fulfilling their legal mandate to act as an interface between science and patient care [2]. Physiotherapy research can contribute to improving quality and clinical risk management at a structural, process and outcome level, resulting in a more efficient use of resources. Research activities in various settings produce significant findings on effective, efficient and safe therapy methods, as exemplified by studies on the long-term physiotherapy care of Parkinson’s patients [53] or the physiotherapy care of patients with knee arthroplasty in acute hospitals or during inpatient rehabilitation [8]. The evidence-based results not only make it possible to avoid overuse, underuse or misuse of patient care, but also show potential, for example, to reduce the length of hospital stay and thus save costs [9], [54].

Based on the findings on collaborative research [55], [56], knowledge synergies could be used to further develop both research and care by establishing and networking physiotherapy research at university hospitals throughout Germany. 

From the profession-specific perspective of physiotherapy, genuine research (at German university hospitals) is part of the professionalization process and significantly enhances the profession in terms of skills and public reputation – e.g. among patients, politicians, health insurance companies and the medical profession [57], [58]. In addition, genuine research expands the research profile of university hospitals and thus establishes career paths, whereby highly qualified personnel can be recruited and retained [19] and the profession can continue to develop [2].

### Macro level

For the healthcare system, there is an opportunity for research findings to be used as a basis for political decisions [59], the creation of new framework conditions and the development of guidelines and evidence-based standards [27], [60]. As part of a learning healthcare system [61], solutions to social challenges such as demographic change, technological progress and multimorbidity [11], [12], [13] can be generated. The solutions offer opportunities and benefits to make the healthcare system more economical and future-proof, for example by developing and implementing cross-sectoral care approaches [27], [62].

In addition to the visions at national level, research in physiotherapy opens up the possibility of international connectivity through the establishment of worldwide cooperation and networks and the fulfillment of recommended guidelines of World Physiotherapy [63], [64].

With regard to the social perspective, the development of physiotherapy research fulfills the ethical obligation of a profession to expand knowledge and provide a service with high social benefit [65]. As a result, the research results contribute to the implementation of mandatory quality assurance (paragraph 135a SGB V) to ensure the best possible, evidence-based care for the population across the board.

## Framework conditions

The following complex, structural, and financial framework conditions at a university hospital or a university respectively can promote the development and expansion of a genuine physiotherapy discipline and genuine research. 

### Structural aspects

Physiotherapy research can take place at university hospitals closely linked to patient care and the medical departments as well as in teaching and research departments at universities. Currently, physiotherapy research is increasingly taking place at university hospitals. However, the proportion of genuine research in the sense of ownership of the entire research process is low. There are a few, very heterogeneously anchored research institutions at university hospitals where physiotherapy research is carried out. At University Hospital Münster, for example, a staff unit for therapy sciences has been set up, while an Institute for Physical Therapy, Prevention and Rehabilitation has been established at Mainz University Medical Center. Within the framework of academic degree courses at the universities, physiotherapy research is just as heterogeneously located. At the University of Lübeck, for example, it is conducted in the Medical Section as part of the degree course “physiotherapy”, at Heidelberg University Hospital in the Department of General Medicine and Health Services Research as part of the degree course “interprofessional healthcare” by a non-physiotherapeutic professorship for nursing science, and at the Charité – Universitätsmedizin Berlin as part of the degree course “health sciences” in the Institute for Medical/Nursing Education and Nursing Science. 

If physiotherapy research is located in interdisciplinary/interprofessional institutions outside of the discipline, this can have the advantage of enabling low-threshold interdisciplinary/interprofessional research. However, the non-specialist location also reduces the visibility of genuine physiotherapy research. The independent, structural location in the scientific pillars, in addition to the care pillar, can create good framework conditions in the medium and long term to ensure the development of genuine research in terms of personnel and funding, e.g. via a separate budget and participation in the internal performance-oriented distribution of funds. As early as 2021, the German Science and Humanities Council called on university medicine to take on the scientific development and professional differentiation of the healthcare professions more strongly as its own task [2]. This is also in the interest of university medicine in order to integrate university-qualified members of the healthcare professions into the development, implementation and management of innovations in healthcare. In the spirit of an integrative model, the German Science and Humanities Council recommends that medical faculties be supplemented by a Department of Therapeutic Science. Similar to medicine, this department should not only be responsible for studies, teaching and research, but also for care areas and the associated staff for example [2], [32].

The German Science and Humanities Council recommends the establishment and promotion of centers for research, teaching and care management as structure-building measures in order to bring together and strengthen existing expertise. Universities and university hospitals should be just as involved as research-intensive universities of applied sciences. These centers serve the development of disciplines and support scientists in early career phases through, for example, adequate job profiles and structured doctoral colleges. They encourage measures for the development and expansion of research in the healthcare professions and promote the development and testing of clinical professorships in the healthcare professions. Research funding measures should focus on the participation of physiotherapy in relevant research projects [32].

### Financial aspects

The centers require adequate funding with permanent positions and reliable structures to accelerate the development of the discipline. Initial funding supports the process, whereby all options for funding the centers should be examined and fully exploited with a view to the social benefits [32]. In addition to permanent positions and reliable structures, external funding is an important item in the financing of research at university hospitals [66]. Applications for external funding for a physiotherapy project can be submitted either by physiotherapists themselves or by professions outside the field. It is important that physiotherapists are perceived as equal scientific cooperation partners. If this is the case, the physiotherapy perspective is taken into account extensively and adequate funding can be applied for the profession. If physiotherapists are in charge of applying for external funding, this can be done as an independent profession or with a non-specialist profession, depending on the institutional anchoring. In both cases, physiotherapy can research genuine questions, physiotherapy resources can be planned and physiotherapy is actively involved in the research project as an equal, scientifically active partner. This forms a good basis for a discourse at eye level.

Academic staff is needed to apply for, plan and carry out high-quality research, and it is often not found promptly or not at all. The current annual academization rate in physiotherapy of 6.1% of physiotherapists [26] as well as the fixed-term employment relationships common in science in accordance with the German Academic Fixed-Term Contract Act [Wissenschaftszeitvertragsgesetz] exacerbate this problem. Clear, structurally anchored career paths at university hospitals in physiotherapy can contribute to increasing the attractiveness of the profession, on the one hand, and be helpful for the development of genuine physiotherapy research, on the other [45]. Bachelor’s graduates are needed as reflective and agile practitioners in patient care for the increasingly complex care situations [22], [48]. In addition, more physiotherapists working at Master’s and doctoral level are needed for research projects in order to develop a genuine scientific discipline, as the Master’s degree and, in particular, the doctorate enable them to independently manage processes of the profession and associated discipline [67]. The low level of university education, which does not cover the whole country, is a burden on professionalization in the therapy professions [20] and consecutively hampers the development of a genuine scientific discipline. 

A higher rate of academization leads to a higher proportion of physiotherapists who are qualified for scientific work. Attractive, preferably permanent scientific employment contracts can in turn bind these highly qualified physiotherapists to university hospitals. In this way, physiotherapeutic and scientific expertise can be used by the university hospitals for sustainably and profitably development. In-house physiotherapy research not only helps to improve patient outcomes and optimize efficiency and cost-effectiveness, but also to increase patient and staff satisfaction as well as staff retention [68].

The recommendation of the German Science and Humanities Council to anchor healthcare professions more firmly at universities includes research and the training of scientists in early career paths [32]. These are prerequisites for expanding the range of academic courses on offer. In addition to the establishment of academic mid-level staff, the consistent development of career paths in university hospitals also includes the sharpening of the physiotherapy research profile. This can be achieved, for example, through post-doctoral programs, habilitation opportunities and the establishment of tenure track professorships for young scientists. The establishment and testing of fully-fledged physiotherapy professorships at university hospitals and/or universities is also crucial. These professorships are associated with the right to freedom of research, freedom to issue instructions and the right to basic equipment. The University of Lübeck is currently the only university location where physiotherapy can be studied at a university in Germany. As they are committed to research and teaching and at the same time have fewer teaching obligations, university professorships are associated with higher research capacities. The testing and implementation of physiotherapy professorships is recommended by the German Science and Humanities Council, particularly at university hospitals [32]. This would be an important stimulus for genuine physiotherapy research and discipline development.

## Current challenges and possible solutions

Genuine physiotherapy research at German university hospitals faces a number of challenges. They need to be overcome if it is to contribute to shaping a sustainable healthcare system. In this section, we look at the professionals and possible implementation strategies.

What is the current situation regarding the professionals needed for genuine physiotherapy research at university hospitals? In order to conduct a survey to determine how many healthcare professionals with a university degree are working at university hospitals, all 35 university hospitals at that time were contacted by the German Science and Humanities Council in 2020. While 15 clinics (approx. 43%) answered the survey, 87% stated that they employed university-educated healthcare professionals [26]. Unfortunately, the study does not record professional affiliation, university degrees such as Bachelor’s or Master’s, the field of activity, the involvement of healthcare professionals in science or their additional tasks alongside clinical care. The actual number of university-educated physiotherapists remains unclear, also due to the low participation in the survey.

The VAMOS Retention Study of Graduates of Physiotherapy Model University Study Programs in North Rhine-Westphalia [69] concludes that 80.0% of employers assign graduates for regular tasks in physiotherapeutic care. Half (51.1%) of graduates are granted partial leave from regular tasks to work on special tasks. These special tasks, which differ from those of persons qualified at a vocational college, include “expert activities for specific technical topics” (38.9%), “concept development, implementation and evaluation” (36.7%), as well as “project work” (35.6%) or “scientific research” (34.4%). 

The recommendation of the German Science and Humanities Council [19], [32] states a rate of 20% of university-educated physiotherapists in the German health care system. In the VAMOS study, 10.7% of graduates stated that they worked full-time or part-time in research and science [69]. It remains unclear if these activities are carried out in research institutes, clinics, authorities or similar institutions. The authors of this paper support the call of the German Science and Humanities Council. In addition to the areas of responsibility mentioned in the VAMOS study, graduates with a Master’s degree can also perform research and clinic physiotherapeutic projects independently [70]. In order to describe the challenges of genuine physiotherapy research more precisely, a specific analysis of the number of physiotherapists involved in research at German university hospitals is necessary. There is currently neither data on personnel capacities for genuine research at German university hospitals nor on their activities. The authors therefore recommend conducting a study on the “involvement of physiotherapists in research at German university hospitals”. From the authors’ point of view, the data from this study would already be a further step towards the implementation of physiotherapists in university research. 

In consensus with the German Science and Humanities Council, the authors recommend adequate funding for scientific personnel capacities and actions to promote scientists in physiotherapy in early career phases [32] as well as the implementation of genuine physiotherapy research at university hospitals.

How can the implementation of genuine physiotherapy research at university hospitals succeed? The four-phase Quality Implementation Framework (QIF) [71], [72] from implementation research can be used for this purpose. In the first phase, the need for genuine physiotherapy research is determined. Furthermore, preparatory work will be carried out to build capacity: university-educated physiotherapists carry scientific competencies considered and used in the university hospital setting. Attractive working conditions are necessary in order to deploy these in university hospitals. Conditions include exemption from clinical activities, time out from clinical work for scientific activities, permanent employment contracts, mobile working, and financial incentives. By implementing the physiotherapy profession in research, university hospitals benefit from the new opportunity to expand their research repertoire and carry out innovative interprofessional research projects. This creates the opportunity, on one hand, to make therapeutic care more efficient. On the other hand, interprofessional and genuine physiotherapy research are a lighthouse and increase university attractiveness with regard to future acquisition of external funding.

**Figure 3:**
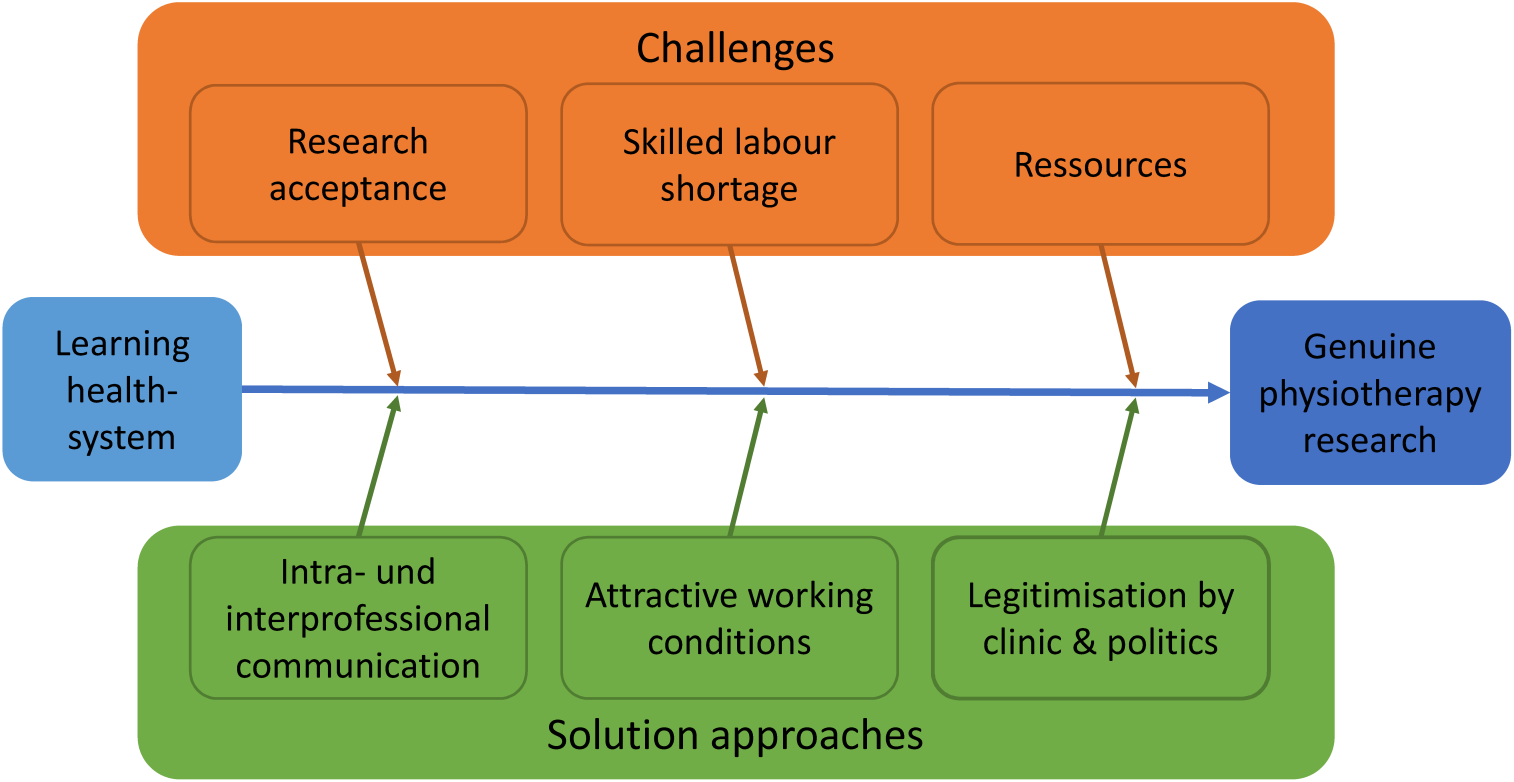
Challenges and proposed solutions for implementing genuine physiotherapy research at university hospitals

In the second and third phase of the implementation process, structures are installed and the actual implementation takes place. This includes university hospitals legitimizing genuine physiotherapy research in the university research portfolio as an official part of the research agenda. Furthermore, the authors propose measures for intra- and interprofessional exchange and cooperation in order to demonstrate practical relevance. The following measures can support this process (Figure 3 (Fig. 3)):

Intraprofessional measures (in the physiotherapeutic team):

- Journal clubs and continuing education
- Integration of trainees in research processes as early as possible
- E-mail newsletters on current developments in research projects including results
- Best practice database (across university hospitals)
- Presentation of current projects from all disciplines in therapeutic team meetings

Interprofessional measures (or other professional groups):

- Conception of interprofessional standards (between healthcare professions)
- Networking with other healthcare professions
- Integration of physiotherapy on the respective ward
- Round table at management level
- Joint discussion and therapy goal planning between doctors, nurses and therapists

The third implementation phase involves consolidating structures and supporting them through coaching and feedback. In the fourth phase, lessons learned from the implementation can be addressed accordingly in the next implementation.

By implementing genuine physiotherapy research in the intra- and interprofessional setting of university hospitals, we are able to make an innovative contribution to overcoming the challenges of the learning healthcare system, including [73], [74]

- multimorbidity (also morbidity expansion),
- demographic change,
- assistive technologies,
- digital applications,
- outpatient care,
- and health behavior.

## Research priorities

In physiotherapeutic research, various fields can be addressed. The trend is moving towards cross-sectoral care, gaining significance due to future developments such as increased outpatient care, shortened hospital stays, and a focus on prevention [75], [76]. Palliative care, taking place both in inpatient and outpatient settings, serves as an example of the necessity for cross-sectoral research.

Current and future areas of action in physiotherapy encompass health promotion, prevention, prehabilitation, as well as curative, rehabilitative, and palliative care. This includes counseling and support services, projects, and local initiatives (e.g., health days), aligning with the goals of public health services [77], [78].

Within these broad research fields, the question of research priorities arises. While recommendations exist for research outside university clinics, research within university clinics and physiotherapeutic research, there are no recommendations that unite all three aspects. The aim was to fill this gap by formulating recommendations for prioritizing physiotherapeutic research at German university hospitals. An initial exploratory research phase was followed by the analysis of various priority recommendations. The final step involved multiple rounds of discussion and feedback to formulate recommendations for prioritizing genuine physiotherapeutic research at university hospitals in Germany. 

Prioritization is crucial due to limited resources (financial, personnel, time). Regarding university hospitals research priorities, the focus lies on improving physiotherapeutic care, taking into account the conditions and interests of a university clinic. Prioritization provides answers for research physiotherapists and funding bodies to the questions: “What is relevant? What has been evaluated already?”

The following provides recommendations for physiotherapeutic research outside university hospitals, first regarding a recommendation for physiotherapeutic research in Germany. In 2010 the Health Research Council [Gesundheitsforschungsrat], the advisory body of the Federal Ministries of Education, Research and Health at that time, commissioned a working group to identify research needs in healthcare professions in Germany. The central goal identified for physiotherapeutic research was to improve the quality of care, requiring fundamental and clinical research, as well as translational, implementation, evaluation, and healthcare research. The increasing chronicity of health issues and the importance of health promotion and prevention were emphasized [27].

Braun et al. provided further insights into research priorities by analyzing physiotherapeutic reports in the German journal physioscience from 2011–2020, comparing them with British priorities due to the lack of empirically based German physiotherapeutic research priorities. Only 21% of publications (16/78) addressed topics found in the respective top ten priorities lists [79]. Hence, research is taking place that does not meet the needs of patients and clinicians. This misalignment highlights the need for national research priorities [79].

Looking abroad and at neighboring disciplines, research priorities were identified and later used to draft physiotherapeutic research priorities at German university hospitals. In England, a Delphi study involving 204 stakeholders from musculoskeletal medicine, neurology, cardiorespiratory medicine, mental health, and wellbeing identified under-researched topics, which were verified through a database search. Topics included optimization, effectiveness, access to physiotherapy, knowledge/experiences/expectations of physiotherapists and patients, patient self-management, diagnostics, and prognosis [80]. A qualitative survey in Switzerland on the perception of physiotherapy research, involving 134 participants (patients, practitioners, researchers, representatives from politics and financing), revealed discussions on physiotherapeutic identity, competencies, visibility, and perception [81]. The research fields mentioned by stakeholders were prioritized in a second step using a Delphi procedure involving 420 participants. Prioritized research areas included physiotherapeutic interventions, assessments, diagnostics, prevention, patient interaction, and education/study. Consideration should also be given to the topics of chronicity and demographic change, with recommendations for multidisciplinary networks to conduct interdisciplinary research and increase their reach [82].

A ranking of research topics was developed from the perspective of public health, considering implementation research, digitalization, translation, and especially research on health professions (e.g., physiotherapy) [83].

**Figure 4:**
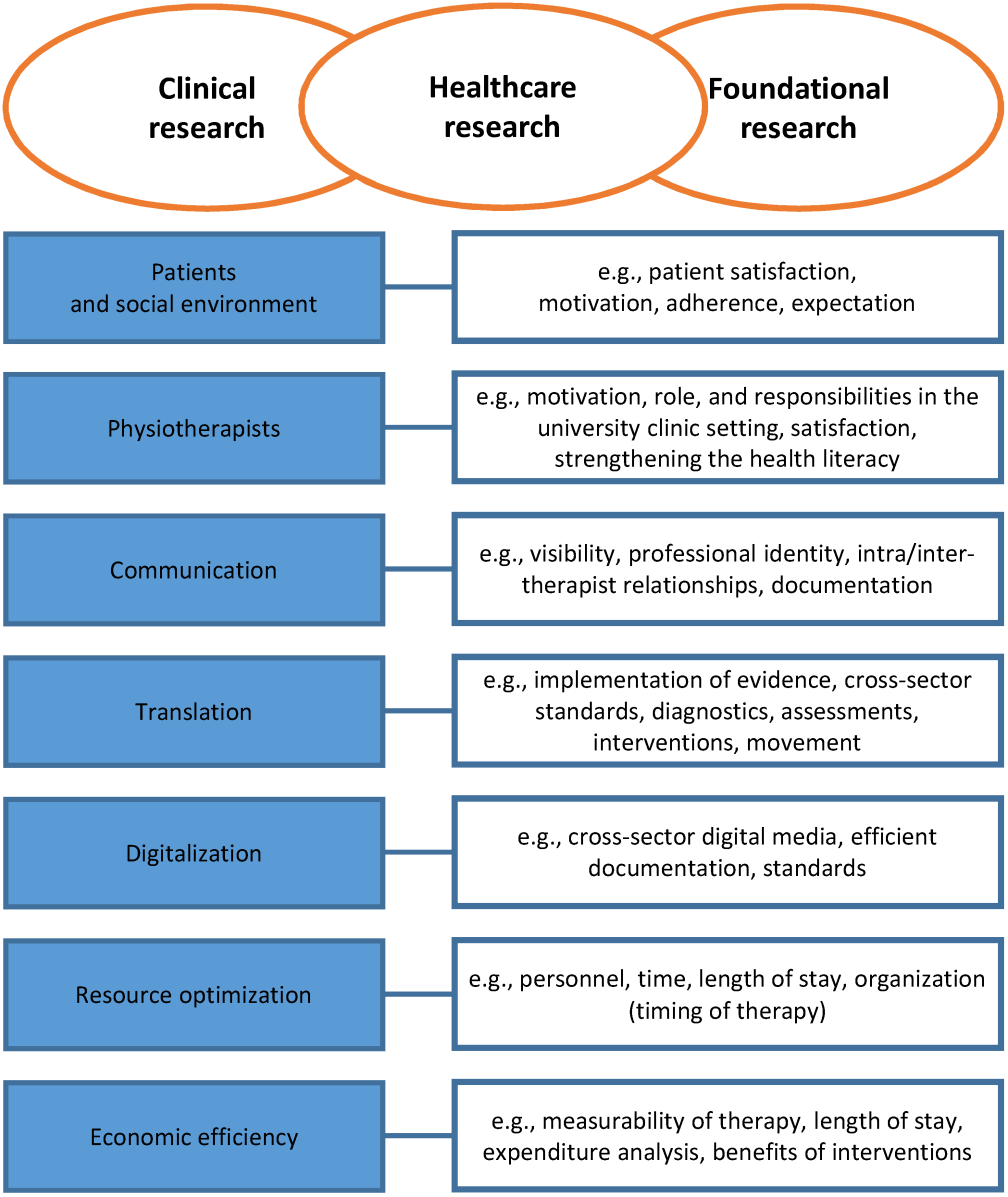
Physiotherapeutic research priorities at German university hospitals

However, the question of which research priorities apply to physiotherapeutic research at German university hospitals remains unanswered. The literature presented above served as the basis for discussions during the scoping workshop. The results of these discussions are depicted in Figure 4 (Fig. 4).

The physiotherapeutic research questions are primarily situated in the field of healthcare research but also serve clinical and foundational research. The overarching research priorities are not hierarchically ordered but should be considered as equally important.

In essence, the mentioned research priorities can be beneficial in increasing the utility of research for physiotherapy and healthcare. Another recommendation is made for a national research agenda and national research priorities.

## Conclusion

University hospitals have a special position at the interface between the scientific and healthcare systems. Physiotherapy at university hospitals increasingly follows the triad of patient care, teaching, and research, but genuine physiotherapy research and scientific discipline formation in particular has not yet reached the recommended level of penetration in relation to university hospitals. Genuine research offers opportunities and benefits to stakeholders at the micro, meso, and macro levels of the learning healthcare system. At the same time, genuine physiotherapy research faces numerous challenges. Framework conditions are needed at all three levels to strengthen physiotherapy research at university hospitals. The development of national research priorities for university hospitals could increase the benefits of physiotherapy research. Further joint efforts and initiatives are needed to establish the emerging physiotherapy profession as an integral part of university hospital research as well as a national and international cooperation partner on an equal footing. With a view to evidence-based patient-centered healthcare, this effort is definitely worthwhile, as physiotherapy plays an essential role in healthcare and can develop its potential even better through genuine research. Genuine physiotherapy research in the university hospitals is therefore not an end in itself, but is at the service of society.

## About the network

In order to jointly strengthen genuine physiotherapy research at German university hospitals, the Network of Researching Physiotherapists at German University Hospitals [Netzwerk forschende Physiotherapeut:innen an den deutschen Universitätskliniken] was founded in the fall of 2022. Every university hospital in Germany can send research physiotherapists to represent it in the network. The network sees itself as the voice and mouthpiece of research-based physiotherapists at university hospitals and the primary point of contact for all stakeholders. Through the association of physiotherapists in research, members can support each other in the development and expansion of physiotherapy research and its infrastructure. The network thus makes a direct contribution to the professionalization of physiotherapy in Germany.

Physiotherapists from 26 of the 36 German university hospitals are currently represented in the network: University Hospital RWTH Aachen, University Hospital Augsburg, Charité – Universitätsmedizin Berlin, University Hospital of the Ruhr-University Bochum, University Hospital Carl Gustav Carus Dresden, University Hospital Düsseldorf, University Hospital Erlangen, University Medical Center Essen, University Medical Center Freiburg, University Medical Center Göttingen, University Hospital Greifswald, University Hospital Halle (Saale), University Medical Center Hamburg-Eppendorf, Hannover Medical School, Leipzig University Hospital, University Medical Center Mainz, Universitätsmedizin Mannheim, LMU Munich Medical Center, University Hospital rechts der Isar, Technical University of Munich, University Hospital Münster, University Hospital Oldenburg, University Hospital OWL, University Hospital Regensburg, Rostock University Medical Center, University Hospital Schleswig-Holstein, University Hospital Würzburg.

## Notes

### Funding

This position statement was developed as part of a scoping workshop held in Hanover from July 5 to 7, 2023, which was funded by the Volkswagen Foundation. The Volkswagen Foundation was not involved at any point in the preparation and publication process of the position statement.

### Competing interests

The authors declare that they have no competing interests
